# Class II MHC Self-Antigen Presentation in Human B and T Lymphocytes

**DOI:** 10.1371/journal.pone.0029805

**Published:** 2012-01-27

**Authors:** Cristina Maria Costantino, Eric Spooner, Hidde L. Ploegh, David A. Hafler

**Affiliations:** 1 Program in Immunology, Harvard Medical School, Boston, Massachusetts, United States of America; 2 Department of Biology, Whitehead Institute, Massachusetts Institute of Technology, Cambridge, Massachusetts, United States of America; 3 Departments of Neurology and Immunobiology, Yale School of Medicine, New Haven, Connecticut, United States of America; Institut Pasteur, France

## Abstract

Human CD4^+^ T cells process and present functional class II MHC-peptide complexes, but the endogenous peptide repertoire of these non-classical antigen presenting cells remains unknown. We eluted and sequenced HLA-DR-bound self-peptides presented by CD4^+^ T cells in order to compare the T cell-derived peptide repertoire to sequences derived from genetically identical B cells. We identified several novel epitopes derived from the T cell-specific proteome, including fragments of CD4 and IL-2. While these data confirm that T cells can present peptides derived from the T-cell specific proteome, the vast majority of peptides sequenced after elution from MHC were derived from the common proteome. From this pool, we identified several identical peptide epitopes in the T and B cell repertoire derived from common endogenous proteins as well as novel endogenous epitopes with promiscuous binding. These findings indicate that the endogenous HLA-DR-bound peptide repertoire, regardless of APC type and across MHC isotype, is largely derived from the same pool of self-protein.

## Introduction

Endogenous peptides presented via class II MHC bind the T cell receptor (TCR) to regulate CD4^+^ T cell development, homeostasis, and activation in the periphery [1]. These peptides represent the majority of MHC-bound ligands displayed by antigen presenting cells (APC) [2] and are derived from a wide variety of endogenous proteins in a diversity of APC-specific stimulatory microenvironments [3].

Common among the APC subtypes are self-peptides presented in course as a byproduct of class II MHC processing. These include the invariant chain (Ii; CD74) chaperone fragment CLIP, which occludes the MHC binding pocket during assembly [4], [5]. Peripheral APC can regulate CLIP expression at the cell surface [6], [7]. Up-regulation of CLIP in tandem with antigenic peptide presentation in activated dendritic cells has been shown to enhance Th1-type cytokine secretion in antigen-specific T cell responders [6]. Although CLIP is the only known non-antigenic self-peptide to elicit a polar shift in the quality of the CD4^+^ T cell response, several groups have reported that self-peptide MHC complexes at the immune synapse stabilize antigenic peptide-TCR interactions and strengthen activating signals [8], [9].

In the absence of antigenic stimuli, endogenous presentation by peripheral APC induces weak, non-specific signaling in CD4^+^ T cells that lowers the threshold of activation in naïve cells [10] and preserves memory cell functionality [11]. A number of these self-peptides, however, act as antigenic epitopes themselves; endogenous presentation is sufficient to activate peripheral CD4^+^ T cells with high-affinity for self-peptide:MHC complexes. Aberrant activation of self-reactive T cells contributes to autoimmune diseases such as multiple sclerosis and type 1 diabetes [12], [13]. Modulation of endogenous antigen presentation in the periphery reduces self-reactivity and prevents the development of autoimmune pathogenesis [14]. Self-antigen specific regulatory CD4^+^ T cells (Treg) also control peripheral immune activation by locally suppressing proliferation and cytokine secretion [15]. Therefore both inflammatory and suppressive reactions can be generated by APC through the regulation of self-peptide generation and presentation to self-specific CD4^+^ T cells.

Although APC do express common self-peptides [16], cell-type specific differences in proteome and lysosomal protease activity can generate unique peptide-MHC repertoires [17]. This is most apparent in thymic epithelial cells, where transcriptional regulation of the cellular proteome results in unique self-peptide expression and presentation that is functionally exploited during thymic selection [18]. In this manner, the cell-type specific proteome is sampled to generate the class II MHC peptide repertoire, and presentation of these tissue-specific peptides dictates APC function.

The relationship between self-peptide identity and APC function may prove to be particularity informative in the case of class II MHC^+^ CD4^+^ T cells. These non-professional APC are thought to present self-peptide exclusively unless loaded with soluble peptide or infected by tropic viruses [19], [20]. The APC function of class II MHC^+^ CD4^+^ T cells remains largely unknown, although several studies have suggested that these cells induce TCR-specific anergy [21]. Indeed, endogenous expression of HSP60 by CD4^+^ T cells has been shown to increase presentation of an HSP60-derived epitope that stimulates HSP60-specific immunosuppression [22]. Cell-type specific presentation of self-antigen in the periphery may therefore have the potential to elicit regulatory responses.

Here, we isolate and identify HLA-DR-bound self-peptides expressed by activated CD4^+^ T cell clones which constitutively express class II MHC. In order to determine the contribution of the cell-type specific proteome to the MHC-bound peptide repertoire, we compared these T cell-derived peptides to HLA-DR-bound peptides isolated from donor-matched B cells. We identified several cell-type specific peptides uniquely expressed and presented by T cells or B cells, including fragments of CD4 and IL-2 and of the B cell receptor heavy and light chains, respectively. Yet despite these cell-type specific differences, we found that the MHC-bound endogenous peptide repertoire was largely shared between T cells and B cells. Both APC types predominately expressed peptides derived from the common proteome. Common peptides presented by these APC had similar frequency of expression, HLA-DR isotype affinity, and, in many cases, identical core sequences. Among these shared sequences, we identified several novel endogenous epitopes. Furthermore, when we compared sequences derived from two donors with unique MHC haplotypes, we found that many of the same proteins and peptide sequences were represented in the peptide repertoire of each. These findings indicate that while the cellular proteome does contribute to unique self-peptide presentation, endogenous peptide expression is promiscuous and largely shared amongst APC.

## Materials and Methods

### Cell culture reagents and antibodies

All cells were cultured in RPMI 1640 medium supplemented with 2 mM L-glutamine, 5 mM HEPES, 100 U/ml penicillin/streptomycin (all from BioWhittaker), 0.5 mM sodium pyruvate, 0.5 mM nonessential amino acids (from Life Technologies) in 25 cm2 vented flasks (CoStar). The media for T cell clones was further supplemented with 5% human AB serum (Mediatech) and 25 U/ml recombinant human IL-2 (Tecin, National Cancer Institute). The media for B cell lines was supplemented with 10% FBS.

The anti- CD3 (UCHT1), CD4 (RPA-T4), CD28 (CD28.2 and 3D10), class I MHC (G46-2.6), class II MHC (Tü39), HLA-DR (L243), HLA-DP (B7.21), HLA-DQ (Tü169), and isotype control monoclonal antibodies were purchased from BD Pharmingen. The monoclonal antibody Tü36, recognizing HLA-DRαβ dimers [23], was purified from hybridoma by Cell Essentials (Boston, MA).

### Generation of CD4^+^ T cell clones and EBV transformed B cell lines

#### Ethics statement

The use of human samples for this study was approved by the Brigham and Women's Hospital Institutional Research Board (IRB).

T cell clones and B cell lines were generated from the peripheral blood of healthy individuals after informed and written consent, as previously described [7]. Briefly, whole mononuclear cells (PBMC) were isolated by Ficoll-Hypaque (GE Healthcare) gradient centrifugation. CD4^+^ T cells were isolated using the CD4+ T cell negative isolation kit II (Miltenyi Biotech) and sorted at one cell per well into 96-well U-bottom plates containing 2×10^5^ irridiated (5000 rad) PBMC, 50 U/ml recombinant Il-2, and 1 µg/ml each anti- CD3 and CD28 (3D10). Clones were expanded for 4 weeks and then subsequently re-stimulated in 25 cm^2^ flasks coated with anti- CD3 and CD28. EBV transformed B cell lines were generated from PBMC as previously described [24].

### Flow cytometry

To confirm purity and expression of class II MHC, T cell clones and B cell lines were stained with fluorophore-conjugated primary antibody (at a concentration of 5 µg/ml). Acquisition was done on a FACScalibur with the CellQuest software (Becton-Dickinson) and data was analyzed using FloJo (TreeStar).

### Mass spectrometry

Cells (10^7^ to 10^8^) were harvested and washed in PBS prior to lysis in buffer containing 1% Triton X-100. Lysates were centrifuged at 15,000×g for 15 minutes at 4°C and pre-cleared once with normal mouse serum and protein A-agarose beads (Pierce) and once with protein A alone. HLA-DRαβ complexes were precipitated with mAb Tü36 conjugated to CNBr-activated Sepharose 4B beads (GE Healthcare). After extensive washing, peptides were eluted with 0.1% trifluoracetic acid. MALDI analyses (Waters Micro MX) was used for initial screening of the eluent prior to further use. Peptides were identified with a Waters NanoAcquity HPLC system operated in a nanoflow mode using reversed phase chromatography. The LC system was directly coupled to a ThermoFisher LTQ linear ion trap mass spectrometer. Analysis was performed using data dependant analysis to maximize the number of peptides subjected to fragmentation. MS/MS data were processed and subjected to database searches using Sequest (ThermoFisher) against the SwissProt databases. Predictions of peptide binding to HLA-DR were made with NetMHCII (http://www.cbs.dtu.dk/services/NetMHCII/) [25].

## Results

### HLA-DR-bound peptide elution from a donor-matched B cell and T cell

We hypothesized that peripheral APC with unique proteomes generate different pools of APC-specific endogenous peptides for presentation via class II MHC. To test this hypothesis, we developed two unique APC types from a genetically identical donor; these are chronically activated CD4^+^ T cell clones and EBV-transformed B cell lines. These APC have similar levels of constitutive class II MHC expression and defined lysosomal protease expression and activity [7], [26]. These APC types also express predominantly self-peptide and have overlapping proteomes with defined singularities.

**Figure 1:**
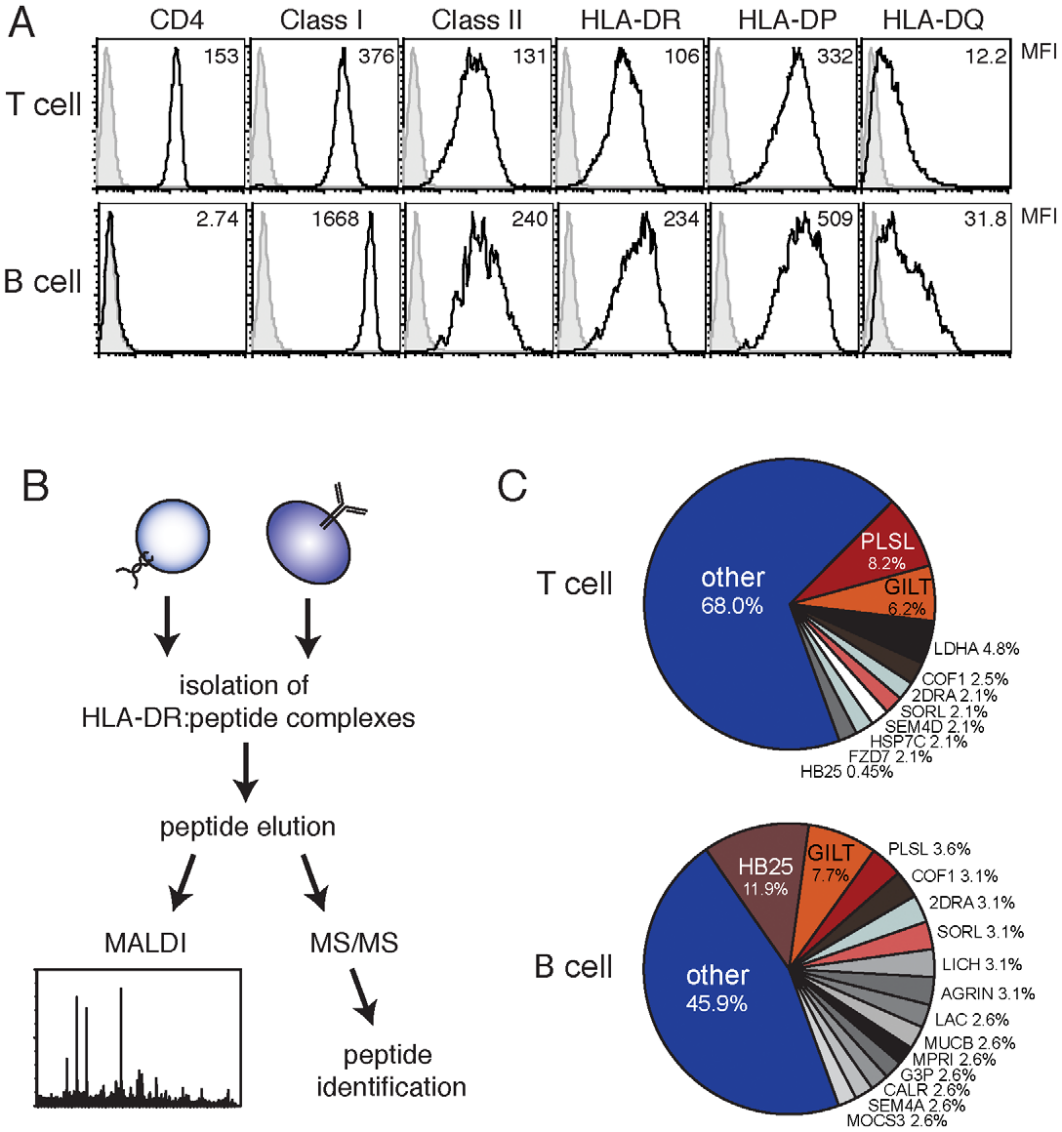
Isolation of HLA-DR:self-peptide complexes from an expanded class II MHC positive T cell clone (T cell) and a EBV-transformed B cell clone from the same donor. **A**. Characterization of class II MHC, class I MHC, and CD4 expression in a representative genetically-matched T cell clone and B cell line. **B**. Strategy used to identify self-peptides. HLA-DR:peptide complexes were immunoprecipitated from T and B cell lysates. Peptides were eluted from the MHC and assessed for quality and contaminants with MALDI before being subjected to multidimensional protein sequencing and identification using the SEQUEST algorithm. **C**. Frequency of common self-peptides identified T and B cells from a donor 1 (DRB1*1301; DRB1*1501 DRB3*0202; DRB5*0101).

We generated and expanded B and T cell APC as described previously [7]. Chronically activated CD4^+^ T cell clones acquire constitutive cell surface class II MHC expression and, like EBV-transformed B cells, can elicit antigen-specific T cell responses when loaded with exogenous peptide [19], [27]. We expanded individual B and T cell clones from a single donor and confirmed cell-surface expression of class II MHC determinants by flow cytometry (**Figure 1A**). We found no significant differences in class II MHC expression between B cell and T cell clones, although the B cells expressed on average higher levels of both class I and class II MHC.

To determine the endogenous peptide repertoire of these cells, we eluted peptides from affinity-purified HLA-DR molecules and performed sequence analysis using liquid chromatography and nano-electrospray ionization tandem mass spectrometry (**Figure 1B**). Peptide preparations subject to MS/MS and peptide identification were verified for quality using MALDI prior to further analysis. Peptide elution was performed in triplicate (for T cells) and duplicate (for B cells) from independent cell cultures derived from a single clone, and was repeated in two donors. We confirmed the specificity of our affinity purification by spiking our lysate with a known peptide prior to immunoprecipitation and elution (data not shown) and by immunoprecipitation with an irrelevant antibody (**Figure S1**).

In total, we identified 791 peptides comprising 384 unique sequences from within the combined B and T cell repertoire (**Table 1** **; Table S1**). We found that 25±5.3% (average ± SD) of the peptides we identified had overlapping sequences and were found in multiple samples, suggesting a bias toward certain peptides in HLA-DR binding, or possibly in our identification protocol. We identified a small number of peptides derived from serum (1.2% of total peptides), primarily derived from serotransferrin and serum amyloid P-component (**Table S5**). While the majority of epitopes we identified were unique, our findings included peptide sequences that are known to bind HLA-DR (DRB1), including sequences from Ii, apolipoprotein B-100, lysosomal acid lipase/cholesteryl ester hydrolase, HLA-DRα, HLA-DRα, cathepsin B, lysosomal protective protein, and phosphoglycerate kinase 1 [6], [28], [29], [30]. We also identified proteins that are presented via class II MHC in other species, including the transferrin receptor, Ig μ chain, and β-galactosidase [31], [32]. These similarities suggest that certain peptides and proteins are more frequently represented in the class II MHC binding pocket than others, regardless of APC type or MHC haplotype. Furthermore, consistent with previous studies [6], [31], we did not find a bias towards transmembrane or endo/lysosomal proteins among the most abundant self-peptides in the binding pool. Taken together, these findings suggest that certain epitopes are globally favored for class II MHC presentation and may be targeted specifically for peptide generation and loading.

**Table 1:** Number and frequency of peptides identified.

	Donor 1		Donor 2	
HLA-DR haplotype	DRB1*1301; DRB1*1501 DRB3*0202; DRB5*0101		DRB1*0101; DRB1*0301 DRB3*0101	
total peptides characterized	644		147	
# peptide sequences	279		105	
peptides sequences with multiple, overlapping hits	82	29.39%	23	21.90%
peptide sequences common to B and T	96	34.41%	4	3.81%
**B cell elution**				
number of cells used for purification	10∧7		10∧7	
total peptides characterized	196		109	
# peptide sequences	81		82	
peptide sequences unique to B cells	48	17.20%^a^	78	74.28%^a^
peptide sequences with multiple hits	25	30.86%^b^	20	24.39%^b^
# of these peptides unique to B cells	9	11.11%^b^	18	21.95%^b^
**T cell elution**				
number of cells used for purification	10∧8		10∧7	
total peptides characterized	448		38	
# peptide sequences	213		30	
peptide sequences unique to T cells	135	48.39%^a^	26	24.76%^a^
peptides sequences with multiple hits	57	26.76%^b^	4	13.33%^b^
# of these peptides unique to T cells	30	52.63%^b^	2	50.00%^b^

a % of total sequences.

b % of cell-type specific sequences.

### Endogenous peptides derived from the common proteome

Of the self-peptides that we identified, a majority was derived from proteins shared within the common proteome of B and T cells. In a single donor (DRB1*1301/1501; DRB3*0202; DRB5*0101), 34.41% of peptide sequences with multiple hits were common to both B and T cells (**Table 1**). The majority of these peptides were derived from six proteins—gamma interferon lysosmal thiol reductase (GILT), HLA-DRα (2DRA), HLA-DQβ (HB25), sortillin-related receptor (SORL1), plastin-2 (PLSL), and cofilin-1 (COF1)—which represented 21.55% and 32.50% of the T and B cell peptide repertoire, respectively (**Figure 1C**).

**Figure 2:**
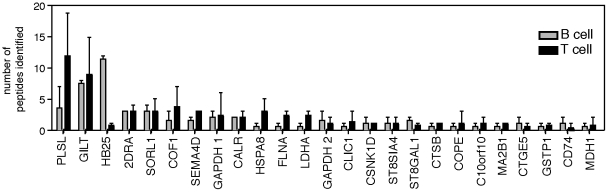
Total number of peptides identified from common proteins in the T cell and B cell self-presentation repertoire. Experiments were performed in triplicate (for T cell clones) and in duplicate (for B cell lines) using cells prepared from donor 1 (mean±range).

Although T and B cells largely presented peptides derived from the same proteins at the same frequency, there was one notable exception. We found that B cells predominantly presented peptides derived from HLA-DQβ (HB25) (11.9%), while T cells presented this protein at a significantly lower frequency (0.45%) (**Figure 1C**). The same core peptide sequence of HLA-DQB1 was identified in both B cell and T cell samples (DVGVYRAVTPQGRPDA; 43–58), so selective use by B cells of a unique peptide sequence cannot explain this discrepancy. Furthermore, such biased presentation was not observed for any other proteins in the shared proteome (**Figure 2**). The increased frequency of this HLA-DQβ peptide in the B cell repertoire could be a consequence of greater HLA-DQβ protein expression (**Figure 1A**); our analysis does not exclude this possibility, although it is interesting to note that peptides derived from class I MHC, which is more highly expressed in B cells than T cells, and HLA-DR or HLA-DP, which both have expression ratios similar to HLA-DQ, were not represented with greater frequency in the B cell peptide repertoire.

We identified several endogenous epitopes that were both shared and abundant in both B and T cells (**Table 2** **; Table S2**). These ‘abundant’ epitopes were classified as peptides containing a shared sequence that were identified in replicate elutions in both a B cell and a T cell sample. Peptides encoding an abundant epitope possessed identical core sequences, each one of which contained the predicted DRB1 p1 binding residues (Y, F, L, I, V, W) followed by 9–14 amino acids. Our data confirms previous reports of epitopes identified from proteins such as HLA-DRα, CD74, and cathepsin B. Additionally, we identified several unique epitope sequences derived from HLA-DRB1, β2-microglobulin, and semaphorin 4D.

**Table 2:** Core sequence of a subset of abundant self-peptides isolated from both T cells and B cells (donor 1).

Protein	Protein Name	Core Sequence	Localization	# Peptides core sequence	Rank
2DRA	HLA class II histocompatibility antigen, DR alpha chain	EFGR**F**ASFEAQG	membrane	15	4
HB2B	HLA-class II histocompatibility antigen, DR beta chain	FLDRYF**Y**NQEESVRFDS	membrane	2	33
HB25	HLA class II histocompatibility antigen, DQ beta chain	D**V**GYRAVTPQGRPDA	membrane	25	3
CD74	HLA class II histocompatibility antigen, gamma chain	DPSSGLG**V**TKQDLGPVPM	membrane	3	24
B2M	Beta-2-microglobulin	KSN**F**LNCYVSGFHPS	membrane	2	35
SEM4D	Semaphorin-4D	NLPDKT**L**QFVKDHPLMDDS	membrane	12	7
APOB	Apolipoprotein B-100	QI**L**PWEQNEQVKNFVAS	secreted	2	35
CATB	Cathepsin B	**F**SVYSDFLLYKSGV	lysosomal	4	21
GILT	Gamma-interferon-inducible lysosomal thiol reductase	F**F**GNGPPVNYKT	lysosomal	42	2
CALR	Calreticulin	**Y**SPDPSIYAYDNFG	cytosolic	10	10
G3P	Glyceraldehyde-3-phosphate dehydrogenase	ENGK**L**VINGNPITI	cytosolic	11	8
		KYDNS**L**KIISNASCTTN		6	13
GSTP1	Glurathione S-transferase	QDGDLT**L**YQSNTILRH	cytosolic	3	25
HSP7C	Heat shock cognate 71 kDa protein	DAAKNQ**V**AMNPTNTVFDAK	cytosolic	10	9

The core sequence represents the largest consensus peptide sequence identified for total nested protein fragments across all samples for a single donor. The total number of peptides identified for donor 1 that contain each core sequence is indicated. Predicted p1 anchor residues for DRB1 binding are in bold (p1 = Y, F, L, I, V, W).

### Endogenous peptides derived from cell-type specific proteome

**Figure 3:**
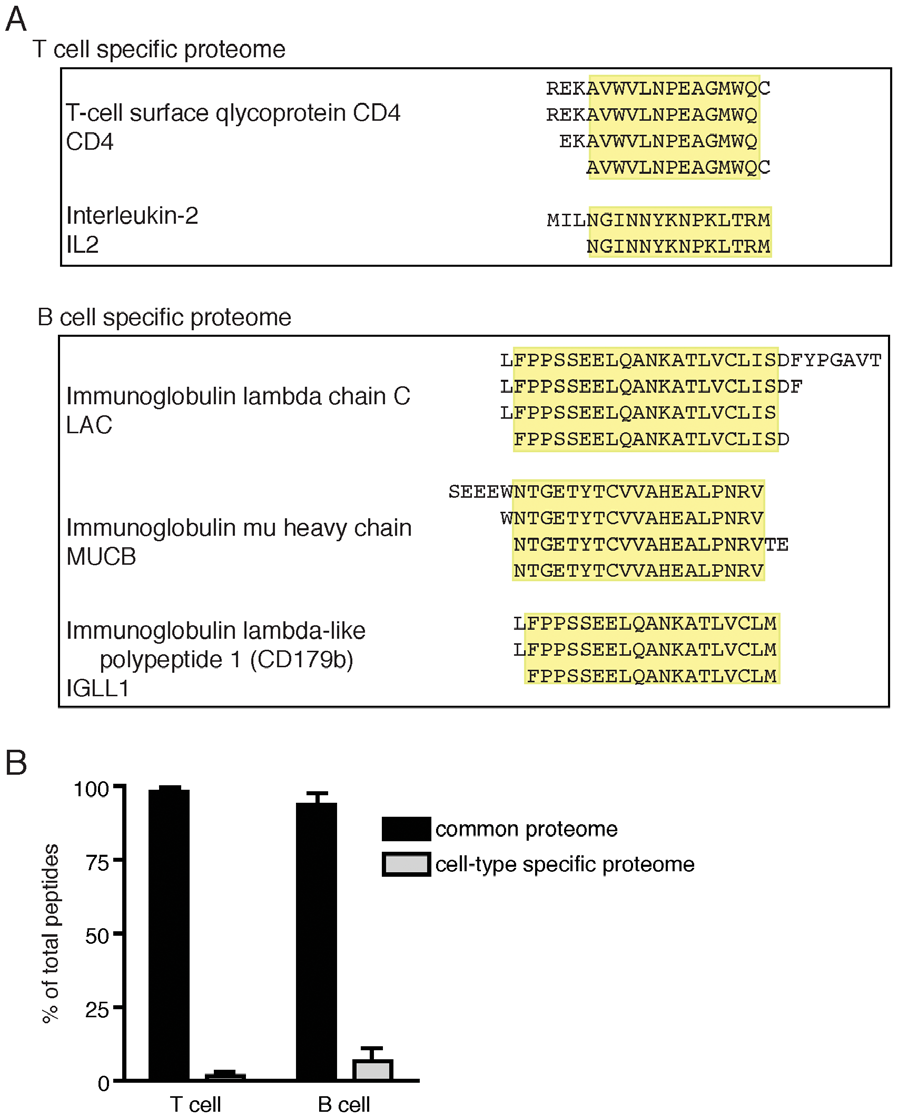
Self-peptides identified from the cell-type specific proteome include key signaling receptors such as CD3, CD4 and the B cell receptor. **A**. Cell-type specific nested peptide sequences isolated from T cells and B cells (donor 1). Predicted core binding sequence is indicated in yellow. **B**. Frequency of peptides derived from the common (on non-cell-type specific) proteome and the cell-type specific proteome in T cells and B cells. Experiments were performed in triplicate (for T cell clones) and in duplicate (for B cell lines) using cells prepared from donor 1 (mean±SD).

In addition to epitopes derived from common proteins, we identified several peptide sequences derived from cell-restricted proteome (**Figure 3**). From our B cell samples, we identified known epitopes in proteins such as immunoglobulin μ heavy chain (**Figure 3A** **, Table S1**). These findings confirm the specificity of our elution protocol and confirm previous observations regarding the capacity for B cells to present tissue-specific proteins [29].

From our T cell derived samples, we identified several novel peptide epitopes that included abundant, overlapping fragments of CD4 and IL-2 (**Figure 3A**). It is likely that these novel epitopes within CD4 and IL-2 are also presented in cells that express CD4, such as monocytes; in cells that can bind and utilize IL-2, such as CD25^+^ B cells and γδ T cells; and in scavenger cells that ingest and present apoptotic CD4^+^ T cells. We also identified peptide sequences, although at a lower frequency, from galectin-1, TGF-β receptor type III, and BDNF/NT-3 growth factor receptor (**Table S1**).

Antigens derived from CD4^+^ T cells injected as a vaccine can activate so called antigen-specific idiotypic and ergotypic responses [33]. HLA-DR^+^ CD4^+^ T cells themselves have been hypothesized to present T cell-derived proteins such as CD25 or HSP60 [22]. Although we did identify an epitope within IL-2 that can be considered “ergotypic” (**Figure 3A**), we were unable to identify any peptides derived from HSP60 in this assay; although we did identify peptides derived from heat shock cognate 71 kDa protein (HSPA8), a ubiquitously expressed chaperone protein. The possibility remains that T cells do possess the capacity to present HSP60 or other idiotypes such as TCRαβ, as the experiments described here lack the necessary resolution to identify rare peptide sequences. Immunogenic epitopes are frequently rare and strong representation in the class II MHC-bound peptide repertoire is not required to activate a T cell response [34], [35].

Overall, we identified very few abundant tissue-specific peptides. Over 90% of peptides identified from both B and T cells were derived from within the common proteome (**Figure 3B**). These data suggest that APC with constitutive class II MHC processing largely sample from the same protein pool and predominantly do not express epitopes derived from cell-type specific proteome, despite high expression of these unique cell type-specific proteins.

### B and T cells utilize MHC isotypes with the same frequency

Several of the epitopes identified in our analysis were found in the binding repertoire of both B and T cells. Given the common identity of these sequences, we hypothesized that B and T APC utilize class II MHC isotypes with the same frequency. Our tissue donor possessed multiple class II MHC alleles, which allowed us to challenge this hypothesis by comparing epitope-MHC affinity across the MHC isotypes for peptides isolated from either B cell or T cell samples.

We used qualitative prediction to generate a matrix of peptide-MHC binding affinities (**Table S3**). In order to determine the binding affinity, we applied the SMM-align method, using default parameter settings on the NetMHCII webserver [25], to each replicated peptide sequence identified in our elution. This method predicted a nine amino acid binding core motif for each MHC isotype and returned a score for binding (IC50 and nM affinity). We judged epitopes with affinity scores less than 500 nM as potential binders, including in our positive scoring both weak (<500 nM) and strong (<50 nM) contacts. For epitopes that contained multiple core motifs, we analyzed all predicted core motif sequences independently and chose for cumulative analysis the sequence with the highest predicted affinity for each isotype. Using this strategy, we broadly surveyed trends in predicted MHC occupancy using our B cell and T cell derived peptide pools.

**Figure 4:**
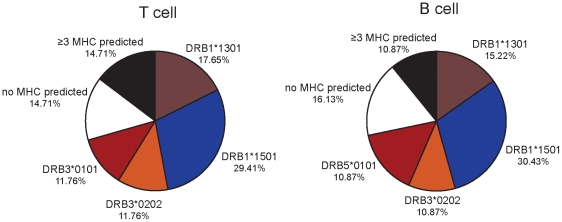
Predicted class II MHC occupancy for self-peptides derived from a single donor with multiple MHC alleles. Peptides with both weak (<500 nM) and strong (<50 nM) predicted affinity are considered as positive for binding. The large frequency of peptides for which no MHC binding could be predicted or which greater than 3 MHC alleles was predicted suggests the likelihood of MHC promiscuity among self-peptides.

We found that predicted class II MHC occupancy was not significantly different for peptides derived from either B cells or T cells (**Figure 4**). Additionally, peptides derived from the APC-specific proteome showed nearly identical isotype binding distribution (data not shown). We found that in total, a third of the peptides we analyzed in all samples scored positive for predicted binding to DRB1*1501. This data may reflect a greater representation of DRB1*1501 over the other isotypes precipitated from our initial lysates.

A significant fraction of peptides we identified, 12.79±2.71% (mean±SD), were predicted to bind more than three MHC isotypes. This finding suggests that the endogenous peptide pool may be highly promiscuous in MHC occupancy. A the relatively large number of amino acids can serve as class II MHC anchor residues, and degeneracy in self-peptide MHC binding has been previously reported [36]. We cannot, however, accurately determine the extent of MHC promiscuity using this analysis strategy. As our prediction determined that 50% of the peptides analyzed bound two or more MHC isotypes, it is likely that this prediction overestimated the extent of degeneracy in MHC binding. Furthermore, this method does not take into account flanking residues or alternative, tertiary binding structure, which can both alter MHC binding affinity [37].

### Self-peptide epitopes shared between genetically diverse donors

We found that B cells and T cells from the same donor sampled largely from the same proteins, and from these proteins the same epitopes, for class II MHC presentation. We next sought to determine whether a unique donor, expressing a different set of MHC alleles, would sample from that same protein pool for presentation. We repeated our APC generation, HLA-DR precipitation, and elution in a second donor that did not express the same set of MHC alleles as the first donor (DRB1*0101, 0301; DRB3*0101) (**Table 1**). Like our first sample set, the gross majority of peptides sequenced were derived from proteins within the common proteome, although we found fewer sequences in common between B and T cells in the second donor (**Table S4**).

We observed a number of peptide sequences in our second donor that were derived from the same proteins that were identified in our first donor (**Table 3**). These included several sequences derived from proteins such as apolipoprotein B-100, β2-microglobulin, and semaphorin-4D. The majority of peptides identified were unique to our second donor, despite their origin from a common protein substrate.

**Table 3:** Self-peptides identified in two donors with unique class II MHC expression.

Protein name	Donor 1 core peptide	Donor 2 core peptide
40S ribosomal protein	KITAFVPNDGCLNFIEEN	FSGVYKKLTGKDVNFE.F
4F2 cell-surface antigen heavy chain	RIGDLQAFQGHGAGN	LKGRLDYLSSLKVKGLVL
Apolipoprotein B-100	QILPWEQNEQVKNFVASH	LSASYKADTVAKVQGV
	TGKIDFLNNYALF	KIEGNLIFDPNNYLPKE
	VYQGAIRQIDDIDVRFQK	
ATP-binding cassette sub-family A member 1	GEKYAGNYSGGNKRKLS	HNVLFDMLTVEEHIWFYAR
Beta-2-microglobulin	RTPKIQVYSRHPAENG	KIQVYSRHPAENGKSNF
	KSNFLNCYVSGFHPS	
Calreticulin	YSPDPSIYAYDNFG	CGGGYVKLFPNSLDQT
Cytochrome P450	AGGPVSVYDASKALTFRMAAR	HAFLPFSGGSRNCIG
Formin-2	EELGARTPRVGGSAHL	EELGARTPRVGGSAHL
Glutathione S-transferase P	**QDGDLTLYQSNTILRH**	**GKDDYVKALPGQLKPFET**
Glyceraldehyde-3-phosphate dehydrogenase	**ENGKLVINGN**PITI	FHGTVKA**ENGKLVINGN**
	**KYDNSLKIISNASCTTN**	**KYDNSLKIISNASCTTN**C
	EGPLKGILGYTEHQVVSS	THSSTFDAGAGIALNDHF
HLA class II histocompatibility antigen, DR alpha chain	**EFGRFASFEA**G	**EFGRFASFEA**QGALANI
Leucyl-cystinyl aminopeptidase	IRDEQYTALSNMPKKSSV	TDKGWSFLLGKYI
L-lactate dehydrogenase A chain	SNPVDILTYVAWKISGFP	NVNIFKFIIPNVVKYSPNC
Phosphoglycerate kinase 1	CGPESSKKYAEAVTRAKQI	PERPFLAILGGAKVADKI
Proactivator polypeptide	EVVAPFMANIPLLLYPQDGPRS	YLPVILDIIKGEMSRPGE
Protein disulfide-isomerase A6	GFPTIKIFGSNKNRPED	DIDLSDVELDDLGKDEL
Semaphorin-4D	NLPDKTLQFVKDHPLMDDS	YIRVLQPLSATSLY
Syntenin-1	LNEEEIRANVAVVSGAPL	NPAILSEASAPIPHD

Peptides with identical core sequences isolated from both donor 1 and donor 2 are indicated in bold.

We also discovered several rare epitopes that shared a peptide sequence between the two donors. Specifically, we identified among these novel sequences in glyceraldehyde-3-phospate dehydrogenase and formin-2 (**Table 3**). Also among the epitopes shared between our two donors was a fragment of HLA-DRα (EFGRFASFEA; 72–81), which was previously identified in an elution from a homozygous DRB1*1501 cell line [29]. Binding of this epitope in both of our donors, one of which does not express DRB1*1501, agreed with our prediction that some endogenous peptides can bind in a degenerate fashion to multiple of the MHC isotypes.

Together these findings indicated that the endogenous peptide repertoire is largely sampled from the common proteome, not only in B and T APC, but also in donors with unique MHC allelic expression.

## Discussion

We initiated this study to identify APC-specific similarities and differences in endogenous peptide presentation. Here we describe, to our knowledge, the largest pool of endogenous peptides identified to date and the first to elute peptide sequences presented from HLA-DR^+^ T cells. CD4^+^ HLA-DR^+^ T cells are novel APC that can generate immunosuppressive rather than activating signals via class II MHC [21]. One third of the potent regulatory CD4^+^ CD25^high^ T cell subset expresses HLA-DR [38], but no studies to date have identified epitopes within the T cell peptide repertoire. We discovered several novel epitopes derived from the T APC specific proteome, including peptides derived from CD4 and IL-2 but no T cell receptor sequences (**Figure 3A**). Many groups have hypothesized the nature of peptides presented by T cells; experimentally predicted epitopes have included fragments of HSP60 [22]. We did not identify any of these predicted ergotypic epitopes in our elution set, but cannot exclude the possibility that the epitopes we identified can elicit an anti-ergotypic response or that other ergotypic epitopes are presented at the cell surface at a low frequency. While antigenic epitopes do not need to be presented at high frequency in order to evoke an immune response [34], [35], we studied the occupation of the majority of HLA-DR complexes in constitutively class II MHC^+^ cells and not the generation of rare antigenic epitopes that may arise as a consequence of activation or other stimulation event due to the limited resolution of our elution protocol.

We eluted peptides bound to HLA-DR in two unique APC with largely overlapping proteomes, B cells and T cells. We found that in both APC, the class II MHC complexes predominantly sampled from the shared endogenous proteome and not the tissue-specific proteome (**Figure 3B**). Indeed, many of the same proteins and peptide epitopes that we here describe have been previously identified in dendritic cell samples with shared allelic expression [6]. Taken together, these data suggest that all HLA-DR^+^ APC possess a similar endogenous peptide repertoire.

While we did not observe many differences in the proteins presented in terms of epitope identity, we did find a significant increase in the presentation of a shared epitope, HLA-DQB1 (43–58). This epitope was represented at a much higher frequency in B cells than in T cells (**Figure 2**). This peptide was previously identified in a peptide elutions using DRB1*1501 B cells [6] and brain tissue samples from patients with multiple sclerosis [39], and is highly abundant in the B cell endogenous peptide repertoire. Why B cells alone selectively express high levels of this peptide remains to be determined. These APC do possess slightly higher levels of HLA-DQ expression than T cells, but similarly high levels expression of other HLA alleles by B cells (**Figure 1A**) does not lead to increased representation in the HLA-DR binding pocket. Exclusive or preferential usage by B cells of the DRB1*1501 isotype could account for this finding, however we did not predict any differences in MHC utilization between B cells and T cells (**Figure 4**). Additionally, selective peptide editing likely does not contribute to differences in DQB1 (43–58) presentation, as this epitope forms stable complexes with DRB1*1501 (DRB2) that are not significantly altered by the enzymatic activity of the loading molecule HLA-DM [40]. The possibility remains that B cells may possess unique mechanisms to enhance presentation of this peptide both *in vitro* and *in vivo*.

In order to determine the impact of MHC allelic expression on endogenous proteome presentation, we extended our analysis to donors with different MHC allelic expression. We found that, even between two donors with unique MHC alleles, the endogenous peptide repertoire was largely derived from the same set of proteins in the common proteome. Most surprisingly, in two genetically distinct donors we identified identical peptide sequences presented in the HLA-DR binding pocket (**Table 3**). Our data indicates that the bulk of endogenous peptide presentation is biased towards the presentation of a few self-proteins. The MHC-bound proteins are sampled from multiple compartments and include both those that are co-localized with antigen presentation molecules (Ii, HLA proteins, membrane and lysosomal proteins), and those that must be targeted to the endo/lysosome (cytosolic and nuclear proteins). These findings are in agreement with coarse analysis comparing thymic and splenic APC in the mouse, which found no significant differences in class II MHC-bound peptides [16]. These common endogenous epitopes may therefore contain sequences favorably suited for stabilization of the T cell synapse [8], [9].

Here we identified a self-antigen repertoire presented by T cells in healthy donors at steady state. As class II MHC expression is upregulated in patients with autoimmune diseases such as multiple sclerosis [41], and during chronic viral infections such as HTLV-1 [42], it would be of great interest to isolate and identify the presented peptide repertoire in T cells derived from these individuals. Class II presentation by these cells can lead to antigen-specific T cell activation, which may contribute to disease pathogenesis [19], [20], [21]. Further work must be done to identify changes in antigen presentation by T cells in chronic disease states and to determine the extent to which any changes in peptide repertoire contribute to CD4+ T cell regulation.

## Supporting Information

Figure S1 **Specificity of peptides eluted from Tu36-bound beads (black) as compared to isotype control (red) with MALDI-TOF analysis.** EBV lysate (0.7 g wet cell mass total) was divided and each fraction was subjected to IP, elution, and mass spectrometry in parallel.
(TIF)

Table S1 **List of Peptides identified in the T cell samples or in the B cell samples from Donor 1.**
(XLS)

Table S2 **Abundant epitopes identified in both the T cell and the B cell peptide preparations from Donor 1.**
(XLS)

Table S3 **Predicted binding of peptides derived from the common T cell and B cell proteome of Donor 1.** Weak binding peptides, in light blue, are classified as those with a predicted affinity of less than 500 nM. Strong binding peptides, in yellow, are those classified as having a predicted affinity of less than 50 nM.
(XLS)

Table S4 **List of peptides identified in the T cell samples or in the B cell samples from Donor 2.**
(XLS)

Table S5 **Non-self peptides identified during protein elution, for both donors.**
(XLS)
