# Supplementary figures and images for: Class II MHC Self-Antigen Presentation in Human B and T Lymphocytes
Source: PLoS One. 2012 Jan 27;7(1):e29805. doi: 10.1371/journal.pone.0029805 (PMC3267721; doi:10.1371/journal.pone.0029805)

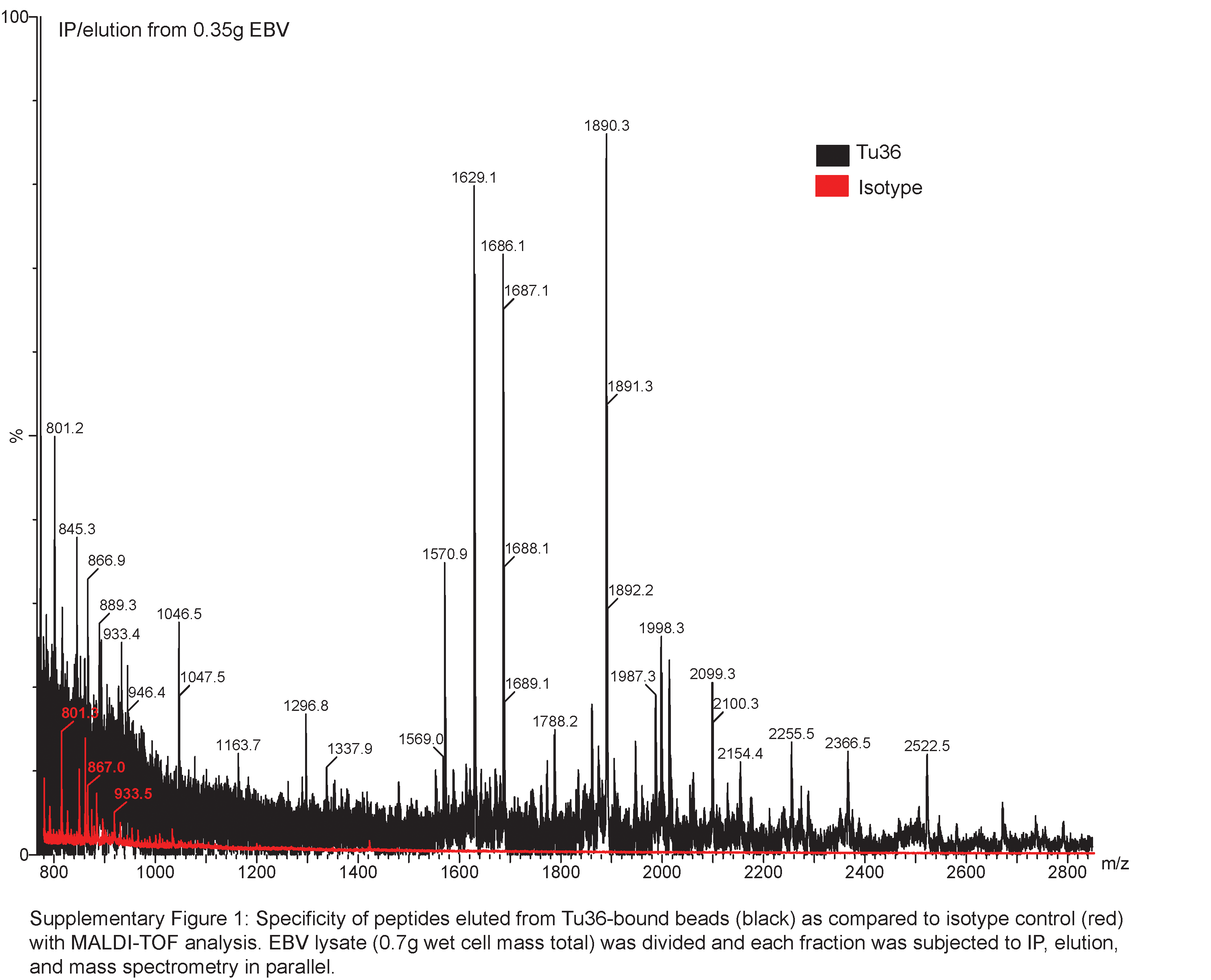

Supplement: Figure S1 — Specificity of peptides eluted from Tu36-bound beads (black) as compared to isotype control (red) with MALDI-TOF analysis. EBV lysate (0.7 g wet cell mass total) was divided and each fraction was subjected to IP, elution, and mass spectrometry in parallel. (TIF) [file pone.0029805.s001.tif]
